# Hold five lines strategy to manage two key points to prevent hospital-acquired infection through ultrasound visualization technology

**DOI:** 10.3389/fmed.2025.1665870

**Published:** 2025-11-26

**Authors:** Ying Wei, Jing Su, Xin Tie, Wanhong Yin

**Affiliations:** 1 Department of Critical Care Medicine, West China Hospital, Sichuan University, Chengdu, Sichuan, China; 2 Visualized Diagnostics and Therapeutics & Artificial Intelligence Laboratory, Institute of Critical Care Medicine Research, West China Hospital, Sichuan University, Chengdu, Sichuan, China

**Keywords:** hospital-acquired infection, critical care ultrasound, point-of-care ultrasound (POCUS), visualization, infection prevention

## Abstract

With advancements in medical technology, the management of critically ill patients has significantly improved. However, the incidence of hospital-acquired infection in intensive care unit (ICU) settings has also increased. These infections not only negatively impact patient prognosis but also waste valuable medical resources. Impaired barrier function and increased local bacterial load, resulting from five primary pathways, are two key points in hospital-acquired infections. Point-of-care ultrasound (POCUS) enables the visualization of secretion accumulation and barrier damage, providing guidance for clinical interventions to restore the barrier and clear microorganisms. This technology also allows for the early detection of potential risks that could lead to hospital-acquired infections, enabling timely preventive measures. Therefore, we propose five-line principles for hospital-acquired infection prevention and control and discuss how POCUS can guide their implementation, aiming to provide a structured reference for clinical practice.

## 1 Introduction

With improvements in support technologies and treatment levels in the intensive care unit (ICU), the incidence of death directly resulting from organ dysfunction has significantly decreased. However, with prolonged hospital stays, ICU patients demonstrate diminished resistance to infections owing to their vulnerable physiological state. Furthermore, extensive invasive procedures and escalating antibiotic use have increased the prevalence of multidrug-resistant bacterial infections, significantly worsening patient prognosis. Therefore, preventing and controlling hospital-acquired infections is pivotal in critical care. It not only determines treatment outcomes but also optimizes healthcare resource allocation and reduces costs (1–5).

Exogenous pathogens proliferate locally after contamination, increasing the bacterial load. If the barrier is concurrently damaged, this can lead to systemic infection, either from these exogenous microbes or from a shift in endogenous microorganisms (6). These scenarios pose a significant threat to patients' health and complicate the clinical treatment and prognosis of hospitalized individuals.

Therefore, hospital-acquired infection prevention and control is a systematic process. First, timely drainage of secretions is carried out to reduce the bacterial load, thereby limiting the growth environment for microorganisms. Second, it is necessary to restore or reconstruct barrier function so that it can resist the invasion of microorganisms. Consequently, we have identified and summarized the five barriers that are most prone to being breached by pathogenic microorganisms to cause infections and have formulated a targeted “hold five lines” bundle to guide treatment.

The implementation of Point-of-care ultrasound (POCUS) helps detect the aggregation of secretions, guides their timely removal, reduces the growth environment for microorganisms and decreases the occurrence of hospital-acquired infection. In light of this, we have conducted a comprehensive review of current research on POCUS and developed an infection prevention and control scheme based on ultrasound monitoring, aiming to provide valuable references and guidance for clinical practice.

## 2 The mechanism and specificity of hospital-acquired infection in the ICU

The occurrence of hospital-acquired infection is a multifaceted pathological process that progresses through various stages. It typically initiates with the colonization of microorganisms at specific anatomical sites within the human body. Under favorable conditions encompassing adequate nutrients, temperature, and humidity, microorganisms promote their proliferation and growth. As this growth advances, their population gradually increases, reaching a substantial concentration referred to as the microbial load.

When invasive procedures or immune compromise weaken mucosal barriers (e.g., in the respiratory tract, skin, or gastrointestinal tract), breaches can occur in this otherwise intact defense system. Exploiting these vulnerabilities created by breached barriers, microorganisms experiencing high-concentration growth can translocate beyond tissue boundaries and ultimately instigate infections that have severe detrimental effects on patients' health and even jeopardize their lives.

Hospital-acquired infection control, which covers various important measures, is the key line of defense for medical safety. First, reducing microbial transmission, such as proper hand hygiene, environmental cleaning and disinfection, and the correct use of protective equipment, is of paramount importance. Second, minimizing microbial colonization and high-density growth within the body can lower local microbial concentrations and weaken the risk of infection through measures such as drainage. Third, reducing damage to mucosal protective barriers requires thorough assessment before medical procedures, adhering to aseptic principles, shortening the duration of invasive operations as much as possible, and promptly removing invasive devices to maintain natural mucosal defense function. Fourth, rational use of antibiotics should be achieved by following drug sensitivity tests for the selection of drugs and standard dosage and treatment duration while strengthening management and education to prevent adverse consequences such as antibiotic resistance or imbalances in bacterial flora caused by misuse. These four aspects work together to build a robust hospital-acquired infection prevention and control system (7).

Among the above measures, reducing the bacterial load and minimizing barrier damage are two areas most likely to be overlooked. We have examined the five typical pathways that are most likely to cause issues in these two areas, summarized the development mechanisms of these five aspects, and analyzed in detail the principles of infection control work on the basis of these five aspects.

**Figure 1:**
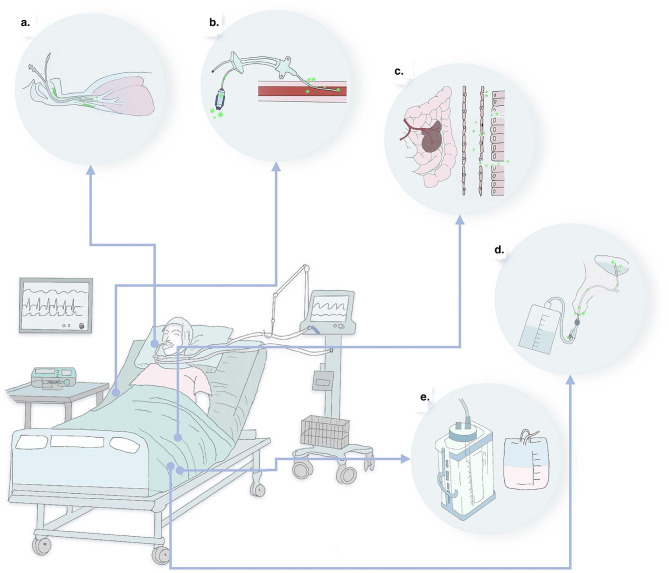
Five major pathways of infection in the ICU result from increased microbial load and barrier disruption. **(a)** Respiratory tract line: respiratory infections are caused by the disruption of respiratory barrier function due to the accumulation of secretions from three sites—pulmonary, oropharyngeal microaspiration and reflux aspiration of gastric contents. **(b)** Intestinal tract line: disruption of the intestinal mucosal–epithelial barrier leads to intestinal bacterial translocation, causing enterogenic infections and sepsis; **(c)** Blood catheter line: direct destruction of the skin and vascular barrier leads to bacterial invasion, resulting in catheter-related local or bloodstream infections; **(d)** Urinary catheter line: placement of a catheter leads to urethral epithelial damage, resulting in urinary catheter-related systemic infections; **(e)** Wound and surgical cavity lines: local and systemic infections caused by the accumulation of secretions in various types of wound areas or surgical zones.

As described in Figure 1, hospital-acquired infections resulting from compromised barrier function in ICU patients can be categorized into five pathways—namely, the “five lines”:

a. Respiratory tract line: in the ICU, the use of invasive endotracheal intubation compromises the integrity of the airway mucosal barrier, resulting in continuous microbial invasion. Typically, infection is characterized by pneumonia caused by damage to the respiratory mucosal barrier due to gastric content aspiration, microaspiration of oral secretions, and prolonged bedridden use of ventilators (8, 9);
b. Intestinal tract line: this leads to intestinal infections due to stress or impairment of intestinal function caused by analgesics or sedatives, resulting in disruption of the intestinal mucosal barrier and bacterial translocation (10);
c. Blood catheter line: this line involves bloodstream infections caused by direct skin and vascular barrier damage during puncture or the formation of bacterial thrombi from long-term catheterization (11, 12);
d. Urinary catheter line: associated with urinary tract and systemic infections occurring as a result of urethral damage caused by urinary catheter insertion (13);
e. Wound and surgical cavity line: this line encompasses infections arising from primary diseases or other causes that lead to infected wound cavities and various types of drainage tubes.

The common characteristic of these infections is the impairment of patients' barrier function, which consequently leads to either endogenous or exogenous microbial infections. Therefore, safeguarding patients' barrier function constitutes the primary measure for preventing hospital-acquired infection in clinical practice.

## 3 How to hold five lines—mechanisms of infection and principles of prevention and control

**Figure 2:**
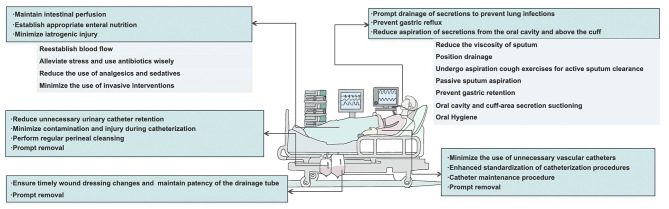
The bundles of hold five lines—principles and measures for the prevention and control of hospital-acquired infection.

With respect to the abovementioned five major categories of hospital infections resulting from barrier dysfunction, we analyzed the mechanisms of the infection sources of these five categories, discussed the principles of hospital infection prevention and control on the basis of the characteristics of each category, as shown in Figure 2, namely, reducing secretion accumulation, eliminating microbial growth environments, restoring barrier function, and formulating a bundle to guide hospital infection prevention and control work in clinical practice.

### 3.1 Respiratory tract line

As patients are mostly in a sedated and bedridden state and require ventilator support for an extended period, pulmonary infections are highly prevalent. For the prevention of hospital-acquired pneumonia (HAP) or ventilator-associated pneumonia (VAP), we propose a “three sites” management approach. This approach aims to prevent the occurrence of pulmonary infections by controlling gastric content reflux, reducing the flow of secretions from the oral cavity (including the paranasal sinuses) and above the cuff through the pharynx into the airway, and addressing inherent infections within the lungs.

#### 3.1.1 Preventing gastric reflux

In critically ill patients, gastric retention is the primary cause of the reflux of gastric contents into the respiratory tract. Owing to haemodynamic instability, it is often necessary to restrict oral intake during this period. When feeding is initiated, there is a propensity for feeding intolerance, which is characterized primarily by elevated residual gastric volume, increased abdominal circumference, bloating, subjective discomfort, vomiting or diarrhea (14). To prevent the occurrence of gastric retention and gastroesophageal reflux after feeding, a comprehensive prefeeding assessment is imperative. Furthermore, modifications should be made to the feeding technique, with strict regulation of eating speed and duration, while vigilantly monitoring for signs of feeding intolerance. Accurate monitoring of gastric tube placement is critical, particularly in cases where high-risk factors are present. In such instances, substituting a nasogastric tube with a nasoenteric tube can be considered a potential strategy to mitigate the risk of reflux.

#### 3.1.2 Preventing microaspiration of oral and endotracheal tube cuff airway secretions

Swallowing function remains intact in healthy individuals, and the occurrence of oral secretions aspirating into the airway is infrequent. However, the majority of critically ill patients are typically sedated, experience impaired swallowing function, and often require tracheal intubation, leading to a high incidence of microaspiration. Therefore, it is imperative to assist in clearing secretions from the oral cavity and above the cuff to minimize the occurrence of microaspiration (15, 16).

The key to preventing microaspiration is to minimize secretion accumulation in the oral cavity and above the cuff. A concurrent goal is to ensure no secretions leak into the airway through the gaps around the cuff. Measures to reduce microaspiration include appropriately elevating the head of the bed; ensuring adequate pressure of the tracheal intubation cuff to minimize lateral inflow; conducting regular oral care—such as routine mouth rinsing, teeth brushing, oral and sinus irrigation, and deep oral secretion aspiration—to control bacterial colonization in the oral cavity; and implementing continuous subglottic drainage and aspiration above the cuff for direct removal of accumulated secretions (17–19).

#### 3.1.3 Reducing the accumulation of lung secretions

To prevent infections caused by pulmonary obstruction in the small and medium airways, as well as the lung parenchyma, ensuring effective drainage of respiratory secretions is crucial. The accumulation of secretions in the airways creates a favorable environment for bacterial growth, resulting in a significant increase in bacterial load and subsequent colonization or infection. Therefore, timely and efficient drainage of respiratory secretions is vital for reducing the risk of bacteria breaching barriers and developing infections (20). Key measures for achieving effective drainage include adjusting sputum viscosity, performing postural drainage for deep-seated secretions, engaging in coughing exercises when necessary, and utilizing passive suction when needed.

Specifically, intravenous or nebulized expectorants can be administered to dilute sputum while maintaining appropriate systemic blood volume (21); positioning techniques, such as lateral decubitus positions (left/right) or prone positions, are employed for dependent lung pneumonia drainage (22); diaphragmatic breathing exercises and nutritional support are provided to prevent weakness and maintain coughing ability; and fiberoptic bronchoscopy may serve as an adjunctive method for sputum clearance when other measures prove ineffective. In clinical practice, patients should first undergo assessment to identify their primary issues so that appropriate measures can be determined on the basis of individual circumstances. Additionally, monitoring lung function is essential for evaluating the effectiveness of a treatment plan and determining its duration.

### 3.2 Intestinal tract line

Although the intestinal tract harbors a vast array of microorganisms, it maintains intact barrier function under normal circumstances to prevent bacterial translocation. However, in the presence of haemodynamic disorders and iatrogenic interventions that lead to mesenteric hypoperfusion and intestinal microcirculation disorders, there is increased apoptosis of intestinal epithelial cells. Consequently, this increases intestinal wall permeability, disrupts the integrity of the intestinal barrier, and ultimately results in bacterial translocation. As a result, enteric bacteria can infiltrate the bloodstream, lymphatic system, and abdominal cavity, thereby inducing systemic pathological changes (23, 24).

The key measure for preventing enterogenous infection lies in maintaining the integrity of the intestinal mucosal barrier while facilitating intestinal peristalsis, restoring adequate blood supply to the intestines, establishing enteral nutrition, and ensuring judicious administration of antibiotics to safeguard and preserve intestinal barrier function. Hemodynamic resuscitation should be promptly initiated to reduce the patient's stress response (25). To ensure adequate intestinal perfusion, the early initiation of enteral trophism or enteral nutrition is recommended to facilitate the restoration of the intestinal mucosal epithelium (26). Furthermore, efforts should be made to prevent iatrogenic factors that may compromise intestinal function, such as excessive analgesia and sedation impeding gastrointestinal motility, and to minimize direct injury caused by abdominal puncture or endoscopic procedures.

### 3.3 Blood catheter line

Vessel catheter-associated infection (VCAI) refers to primary infections occurring during catheter placement and within 48 h after removal, which are specifically unrelated to infections in other areas. This includes both local infections at the catheter site and bloodstream infections, known as catheter-related bloodstream infection (CRBIS). Central venous catheters are the most commonly used and vulnerable type of catheter for infection in ICU settings. Controlling these types of infections involves three key aspects: assessing proper placement, employing meticulous procedural techniques, and implementing vigilant monitoring during retention periods. Direct mechanical damage caused by puncture disrupts the vascular barrier of the skin, significantly increasing the risk of infection. Therefore, reducing unnecessary retention of vascular catheters is a primary measure to prevent CRBSI.

The use of chlorhexidine disinfection, sterile surgical gowns during puncture procedures, and large surgical drapes can significantly reduce infection rates. However, controlling the mechanical complications associated with punctures remains a challenge, necessitating enhanced efficiency in this process (27, 28). During catheter retention procedures, regular skin disinfection and dressing changes should be performed, and surveillance for thrombosis around the catheter insertion site should be intensified. If any thrombus formation is detected, if a catheter has reached its expiration date or is no longer necessary for patient care purposes, it should be promptly removed to mitigate the risk of infection.

### 3.4 Urinary catheter line

Given the imperative need for strict fluid intake control among ICU patients, along with the widespread utilization and simplicity of urinary catheterization procedures, there is a considerably high prevalence of urinary catheter usage. Like vascular catheters, urinary catheters breach barrier function. Therefore, minimizing unnecessary retention time is crucial for preventing associated infections (29). Standardizing disinfection of the perineal region poses challenges due to its anatomical characteristics. Therefore, proper perineal cleansing with gentle techniques during urinary catheter insertion is crucial to minimize contamination and damage to the urethral epithelium (30, 31). Regular perineal cleansing as part of daily care greatly reduces the incidence of urinary tract infections. Once patients no longer require strict fluid monitoring, timely removal of the catheter must be carried out to minimize its overall duration of use.

### 3.5 Wound and surgical cavity line

The key to preventing wound and surgical area infections lies in active drainage and timely evaluation for the early removal of the drainage tube. While it facilitates local fluid and pus discharge, reduces the risk of infection, and promotes surgical wound healing, the drainage tube may also serve as a medium for bacterial invasion and colonization. Ensuring unimpeded initial drainage is imperative to minimize the potential source of infection. Additionally, proactive disinfection and sterile dressing coverage at the drain site are essential to prevent local skin infections. Monitoring accumulated materials within the cavity should be intensified. If there is a significant reduction in accumulation or poor drainage after assessment, the drain should be removed early.

## 4 The significance of POCUS information

The use of POCUS in critical care is invaluable because of its multidimensional monitoring capabilities, which enable real-time visualization of anatomical structures, functional changes, blood flow, and pathological variations. Proficiency in critical care ultrasound techniques allows clinicians to significantly increase monitoring efficiency (32). Furthermore, the point-of-care nature of critical care ultrasound provides doctors with essential physiological information about patients in real time, which is crucial for analyzing their condition and formulating treatment plans. Like visual stethoscopy, ultrasound has become an integral tool in clinical diagnosis and treatment processes, guiding therapy decisions. Harnessing the advantages of POCUS in ICU infection control processes on the basis of the “five lines” framework can significantly improve management precision.

## 5 Five-line scheme with POCUS information

**Figure 3:**
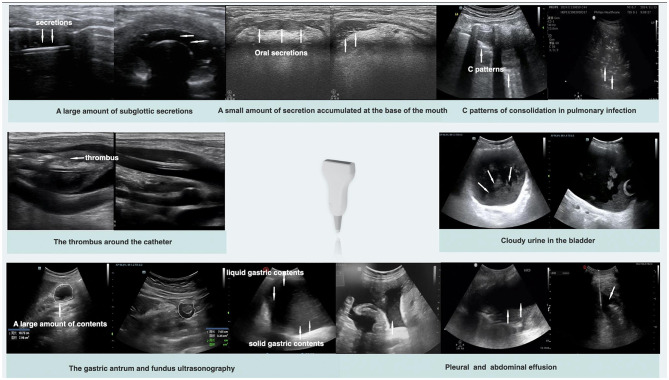
Typical ultrasound images of the five lines.

Given the aforementioned characteristics of ultrasound visualization technology, POCUS plays a significant role in infection control within healthcare settings. As shown in Figure 3, it enables direct observation of the accumulation of infectious secretions, evaluation of gastrointestinal and other organ functions, monitoring of pulmonary atelectasis, and assessment of the degree of barrier disruption. These capabilities make ultrasound an invaluable tool for implementing infection control measures, enabling real-time, dynamic adjustments to treatment plans and improving both the effectiveness of infection control and the precision of therapeutic interventions (33).

### 5.1 Respiratory tract line

#### 5.1.1 Gastroesophageal reflux monitoring

POCUS monitoring of gastric retention is invaluable for the early detection of gastrointestinal dysfunction, facilitating timely intervention and reducing the occurrence of reflux. Before initiating enteral feeding, a routine assessment of gastric antral contraction can serve as a predictive tool to determine the likelihood of feeding intolerance, thereby enabling informed decisions regarding the initiation and adjustment of feeding rates (34). For low-risk patients during the feeding process, monitoring before and after adjusting the feed dosage or composition is usually sufficient. High-risk patients prone to reflux aspiration require more frequent monitoring to adjust feeding rates on the basis of measurements such as gastric antrum size and residual volume. If necessary, feeding must be promptly discontinued to prevent reflux aspiration (35–37). Additionally, for patients with frequent episodes of gastric paralysis, simultaneous monitoring of the gastric fundus should be conducted to promptly detect instances of delayed gastric emptying (24, 25).

#### 5.1.2 Observing the secretions orally and the cuff used for tracheal intubation

The oropharyngeal and tracheal tube cuffs are potential sites for the accumulation of secretions. Timely drainage of oral and cuff secretions is essential to prevent microaspiration. Although tests such as α-amylase and gastric protease are available for detecting microaspiration from the mouth and stomach (38), visual tools remain essential for the swift evaluation of suction indications and effectiveness, particularly when patients cannot cooperate with mouth opening. POCUS enables real-time visualization of secretion accumulation at the base of the tongue, facilitating guidance during suctioning procedures. Similarly, the assessment of airways in intubated patients offers clear visualization with high sensitivity and specificity for detecting secretion accumulation below the vocal cords, significantly improving management efficiency and reducing lung infections caused by microaspiration (18, 39).

#### 5.1.3 Examine the state of pulmonary infection

Although X-rays and CT scans are commonly used to evaluate pulmonary infections, chest radiographs provide limited clinical information because of their two-dimensional nature. Additionally, performing a CT scan can be burdensome for critically ill patients, as it provides only static images, making dynamic observation inconvenient. Consequently, bedside lung ultrasound is increasingly used in intensive care unit (ICU) settings for real-time, dynamic assessment of pulmonary conditions (40, 41).

As discussed, the increased microbial burden resulting from prolonged immobilization is a pivotal factor predisposing critically ill patients to infections. Protocol-driven ultrasonography (e.g., BLUE or FALLS) can systematically monitor real-time debris accumulation across different positions. This guides clinicians in optimizing the timing for pulmonary drainage (42, 43). Using 12 pulmonary ultrasonography zones, clinicians can achieve a comprehensive three-dimensional assessment of the distribution of lung lesions and determine their severity on the basis of different phenotypes. Typically, the lungs exhibit an A-pattern. However, when secretions begin to accumulate, a B-pattern often initially appears in the dependent areas of both lungs, where gas–liquid mixing occurs within the alveoli. If the condition worsens, a C-pattern will manifest. On the basis of the different manifestations in various zones, the overall pulmonary condition can be assessed and managed accordingly. When ultrasound reveals infections primarily distributed in the posterior fifth and sixth zones with evident gravity dependence, active positional drainage techniques, such as prone positioning, should be implemented. However, if patients exhibit local or unilateral changes, bronchoscopy is required to examine specific sites for tracheal abnormalities. An evaluation of diaphragm thickness and contraction rate becomes necessary, along with appropriate nutritional support and diaphragmatic exercises, when patients demonstrate an impaired ability for autonomous sputum expectoration (personal communication).

### 5.2 Guiding the restoration of intestinal perfusion and establishing an intact intestinal barrier

The use of POCUS to protect the intestinal mucosal barrier and prevent enterogenic infections involves three key aspects: assessing intestinal perfusion, establishing enteral nutrition, and evaluating the impact of analgesia and sedation. POCUS allows direct observation of arterial blood flow in the mesentery to evaluate the presence of acute or chronic ischemia in the intestinal mucosa (44, 45). A significant decrease in blood flow may suggest abnormalities such as mesenteric vascular thrombosis. When necessary, mesenteric angiography can be performed to assess the intestinal perfusion status accurately and determine whether enteral nutrition can be initiated (25). Additionally, regular monitoring of changes in bowel diameter, folds, wall thickness, and stratification during enteral nutrition is essential to evaluate intestinal functional status and prevent enterogenic infections related to feeding. Analgesia and sedation should be adjusted as necessary to prevent infections on the basis of assessments of bowel movement patterns and content conditions.

### 5.3 Assisting in the placement of vascular catheters and performing monitoring

In the ICU, ultrasound guidance can effectively reduce hospital-acquired infections during catheter placement and maintenance procedures. Owing to the need to administer high concentrations of fluids or specialized infusions, ICU patients often require central venous access. In addition to conventional sites, such as the internal jugular and subclavian veins, ultrasound-guided peripherally inserted central catheter placement can minimize damage to major blood vessels and avoid unnecessary central venous catheterization attempts. POCUS guidance during catheter insertion significantly reduces the number of puncture attempts and minimizes the risk of skin and vascular injury.

While some studies suggest an increased risk of contamination with ultrasound guidance, this is primarily due to improper aseptic techniques. Therefore, strict adherence to the previously mentioned infection control principles should be emphasized when ultrasound guidance is used (28, 46). Prompt identification and removal of thrombi via POCUS are effective measures to prevent catheter-related bloodstream infections, as thrombi around the catheter frequently lead to septic emboli formation, creating a detrimental cycle (47).

### 5.4 Assisting in the diagnosis of urinary tract infections

POCUS provides objective information on bladder structure and content, offering precise support for urinary catheter management and urinary tract infection (UTI) risk assessment that surpasses conventional methods.

#### 5.4.1 Guiding catheter insertion and retention decisions

Prior to catheterization, bladder ultrasound can accurately measure post-void residual urine volume. Avoiding unnecessary catheterization when the residual volume is < 400 mL and the patient shows no symptoms of urinary retention can reduce the risk of infection at the source. Studies have confirmed the high accuracy of ultrasound and dedicated bladder scanners (48, 49). For difficult catheterizations (e.g., in male patients with benign prostatic hyperplasia), real-time ultrasound guidance can assist in localization, reducing urethral mucosal damage caused by repeated attempts, as mucosal injury is a significant risk factor for bacterial colonization (50).

#### 5.4.2 Dynamic monitoring of infection risk and guiding catheter removal

POCUS provides objective indicators that can signal infection risk earlier. Sediment/debris within the bladder lumen is an important predictive indicator. Research shows that the sonographic finding of echogenic debris in the bladder is significantly associated with positive urine cultures, particularly in pediatric populations (51, 52). Even with clear-appearing urine, the presence of substantial debris suggests a high risk of infection, necessitating enhanced intervention. Furthermore, bladder wall thickness can serve as a reference indicator; chronic inflammation or infection can lead to diffuse bladder wall thickening, and changes in thickness can be used to assess treatment response (50).

#### 5.4.3 Investigating causes of complex urinary tract infections

POCUS effectively identifies anatomical factors leading to refractory infections. In children with febrile UTIs, ultrasound can assess renal echogenicity and abnormalities such as vesicoureteral reflux. Additionally, hydronephrosis, bladder diverticula, and calculi are key targets for ultrasound screening. Identifying these conditions helps in formulating targeted treatment strategies rather than merely addressing the catheter itself (53).

### 5.5 Wound and surgical cavity monitoring

The core value of ultrasound in wound and surgical cavity management lies in its ability to non-invasively and real-time “see through” subcutaneous and deep tissues, facilitating a shift from empirical drainage to image-guided precision management.

#### 5.5.1 Precise quantification and characterization of effusions

POCUS not only detects fluid collections but also estimates the volume of abscesses or effusions by measuring dimensions and applying formulas like the ellipsoid method, providing an objective basis for deciding on drainage. By analyzing echogenicity, ultrasound effectively differentiates the nature of the fluid: anechoic suggests simple serous fluid; hypoechoic or heterogeneous echoes suggest pus or necrotic tissue (abscess); floating punctate echoes may indicate hematocele or viscous infected fluid. This qualitative assessment is crucial for therapeutic decisions (conservative observation, percutaneous drainage, or surgical debridement) (54, 55).

#### 5.5.2 Guiding percutaneous puncture and drainage

For fluid collections requiring drainage, POCUS is the preferred guidance tool. It can display the needle path in real-time, allowing the operator to avoid important blood vessels and organs and enter the center of the fluid cavity via the safest and most effective route. This method significantly improves the success rate and reduces complications, as demonstrated in scenarios such as abdominal paracentesis and facial abscess drainage (56, 57).

#### 5.5.3 Optimizing drainage tube management

After surgery or tube placement, POCUS can immediately confirm whether the drain tip is located within the target fluid collection. During the drainage process, periodic ultrasound re-examination dynamically monitors the reduction of fluid volume and cavity collapse. The near-disappearance of the fluid collection on ultrasound serves as a strong indicator for drain removal, being more reliable than solely observing drainage output because it can detect localized or multiloculated residual collections. Furthermore, POCUS can early identify causes of inadequate drainage, such as tube blockage or the formation of new encapsulated collections, thereby guiding timely clinical adjustments (54, 58).

## 6 Discussion

In this review, we have systematically detailed the five most common pathways for HAIs in the ICU and proposed a structured “hold five lines” prevention strategy centered on visualization technology. The innovation of our approach lies in positioning POCUS as a multimodal tool for assessment and ongoing management, deeply integrating it into every facet of infection control to enhance and optimize traditional care bundles.

Established infection prevention bundles, such as those for VAP and CRBIS, have effectively reduced infection rates through standardized protocols and remain the cornerstone of ICU care. However, the execution of these bundles is often based on pre-defined schedules and indirect clinical signs, lacking direct insight into the patient's real-time and individualized pathological status. Our “five-line” strategy aims to bridge this gap by incorporating POCUS. The unique value of POCUS lies in its ability to provide multidimensional anatomical, functional, and hemodynamic information. This capability advances infection control from standardized protocols to image-guided precision management. For instance, in managing the “respiratory tract line,” POCUS not only assesses lung consolidation and secretion retention using A-/B-/C-line patterns to guide postural drainage and bronchoscopy but also directly visualizes subglottic secretions, transforming scheduled suctioning into an evidence-based, targeted intervention (39, 40). This integration of anatomical imaging with functional assessment is the core manifestation of POCUS as a multimodal tool in the ongoing care of patients.

The multimodal role of POCUS makes it a powerful asset for the continuous management of ICU patients. It moves beyond mere diagnosis, enabling a closed-loop process of assessment-intervention-re-assessment. In managing the “intestinal tract line,” POCUS provides critical decision-making support for initiating enteral nutrition, adjusting infusion rates, and assessing gut perfusion under vasoactive support by evaluating gastric motility, residual volume, and mesenteric artery flow (36, 37). For vascular lines, POCUS not only optimizes puncture paths during insertion to minimize trauma but also monitors for catheter-related thrombosis during retention, allowing for pre-emptive intervention before infection escalates (33, 47, 59). This integrated, dynamic bedside monitoring is unmatched by other single-modality imaging techniques and aligns with the trend toward precision and personalized critical care.

Despite its considerable advantages, the application of POCUS has non-negligible limitations. Primarily, its accuracy is highly operator-dependent. Both image acquisition quality and interpretation accuracy rely heavily on the operator's training and experience, which can lead to inter-operator variability (60). Secondly, variability in resource availability presents a global challenge. The availability of ultrasound machines and the establishment of sustainable training and quality assurance programs can be difficult to achieve in resource-limited settings (61, 62). Furthermore, POCUS has inherent technical limitations; acoustic windows can be compromised by bone and gas, leading to poor image quality in specific situations, which can hinder diagnosis. Finally, POCUS findings are sometimes non-specific for infection and must be integrated with clinical context and other tests to avoid over-interpretation.

Looking forward, efforts should focus on standardizing and disseminating POCUS training and developing standardized scanning protocols and interpretive criteria for specific infection control scenarios. Exploring the integration of POCUS with artificial intelligence to assist in image analysis and quantification is a promising direction to mitigate operator dependency (63–65). Furthermore, more prospective studies are needed to validate the actual impact of integrating POCUS into the “five-line” strategy on patient outcomes, including HAIs incidence, antibiotic use duration, and mortality.

In conclusion, the “hold five lines” strategy, deeply integrated with POCUS, represents a more proactive and precise paradigm for infection control. By fully leveraging the strengths of POCUS as a multimodal tool while acknowledging and actively addressing its limitations, we can build a more robust and visualized defense line against HAIs for critically ill patients.
