# Correction: Hold five lines strategy to manage two key points to prevent hospital-acquired infection through ultrasound visualization technology

**DOI:** 10.3389/fmed.2025.1764844

**Published:** 2026-01-12

**Authors:** Ying Wei, Jing Su, Xin Tie, Wanhong Yin

**Affiliations:** 1 Department of Critical Care Medicine, West China Hospital, Sichuan University, Chengdu, Sichuan, China; 2 Visualized Diagnostics and Therapeutics & Artificial Intelligence Laboratory, Institute of Critical Care Medicine Research, West China Hospital, Sichuan University, Chengdu, Sichuan, China

**Keywords:** hospital-acquired infection, critical care ultrasound, point-of-care ultrasound (POCUS), visualization, infection prevention

The order of authors in the author list of the published paper was erroneously given as:

Wanhong Yin^1, 2*^, Ying Wei^1^, Jing Su^1^ and Xin Tie^1^

The correct author list reads:

Ying Wei^1^, Jing Su^1^, Xin Tie^1^ and Wanhong Yin^1, 2*^

The original version of this article has been updated.

